# Hierarchical Variance Analysis: A Quantitative Approach for Relevant Factor Exploration and Confirmation of Perceived Tourism Impacts

**DOI:** 10.3390/ijerph17082786

**Published:** 2020-04-17

**Authors:** Quang Hai Truong, An Thinh Nguyen, Quoc Anh Trinh, Thi Ngoc Lan Trinh, Luc Hens

**Affiliations:** 1 Institute of Vietnamese Studies & Development Sciences, Vietnam National University (VNU), Hanoi 10000, Vietnam; truongquanghai.ivides@gmail.com; 2 VNU University of Economics and Business, Vietnam National University (VNU), Hanoi 10000, Vietnam; 3 VNU University of Science, Vietnam National University (VNU), Hanoi 10000, Vietnam; trinhquocanh@hus.edu.vn; 4 School of Economics and Management, Hanoi University, Hanoi 10000, Vietnam; trinhngoclan181@gmail.com; 5 Vlaamse Instelling voor Technologisch Onderzoek (VITO), Boeretang 200, 2400 Mol, Belgium; luchens51@gmail.com

**Keywords:** hierarchical variance analysis, residents’ perceptions, socio-demographic variables, ANOVA, linear regression model, perceived tourism impacts, overall impact assessment, Ly Son Island, Vietnam

## Abstract

The issue of tourism impacts is one that has plagued the tourism industry. This study develops a quantitative approach using hierarchical variance analysis, which deals with the exploration of the relevant factors and the confirmation of their significant contribution to analyze the residents’ perception of tourism impacts. Hierarchical variance analysis includes three mathematical procedures: Cronbach’s alpha tests, the exploration of relevant factors, and a hierarchical factor confirmation. Data are collected using a structured questionnaire completed by 452 surveyed residents living in Ly Son Island, Vietnam. The significant effects of socio-demographic variables on the overall impact assessment are observed. The bilateral and simultaneous relationships are analyzed using a one-factor ANOVA. A two-factor ANOVA shows the significant contribution of each socio-demographic variable on the economic, socio-cultural, and environmental impacts. Interaction between factors such as “Education level”, “Type of work”, etc. are hierarchically confirmed. The findings allow a better understanding of the residents’ perception of the effects of tourism on society, the economy, and the environment. This provides a scientific basis to help define problems and promote legal regulations for community participation in tourism planning in a small island destination.

## 1. Introduction

Tourism development impacts the local economy and residents’ lives socially, economically, and environmentally [1,2]. A stakeholder approach is used to assess the impacts of tourism [3,4]. Local residents link tourism development with the challenges of sustainable development, which affect their support for further tourism development [5,6], more hospitality, and for the sustainability of tourism [7]. Perceived environmental pollution reduces the support of residents for tourism development [5]. Perceived economic benefits increase the support of residents towards tourism development [8,9,10,11] because it provides opportunities for employment for local residents in general, and for women in particular. This enables them to be more independent, raises local land prices [1], and provides increased income opportunities [11,12]. Perceived social impacts, such as changes in the life styles and customs of local residents, decrease local social moral standards [1]. The perceived environmental impacts include generating municipal solid waste and carbon dioxide, degenerating the quality of the environment, disturbing the regular life of residents, and destroying the peaceful character of villages [1,13,14]. Perceptions differ between households on the socio-demographic characteristics and on the stages of local tourism development [7,15,16]. A better balance between the socio-economic impacts and environmental considerations in residents’ perceptions is needed [5].

## 2. Literature Review

Residents’ perception of tourism impacts has been quantitatively studied in depth. Combining socio-psychological theories with mathematic models such as Cronbach’s alpha tests, Fisher test, ANOVA, Structural Equation Modeling (SEM), multi-level factor, and regression analysis provides theoretical and practical insights allowing us to understand the impacts of the stressors and beneficiaries in tourism communities [17,18,19,20,21]. A series of aspects of residents’ support towards tourism development are quantified using a triple bottom line approach [22] involving the following: a two-dimensional informedness–involvement tourism grid [23], self-perception theory [24], social exchange theory [25,26,27,28], social exchange theory combined with identity theory [29], and the cognitive appraisal theory [30]. Tourists’ safety is analyzed by the Rimal and Real’s risk perception attitude framework [31]. The item response theory measures the sustainability perception of residents [32]. The du Cros model assesses tourism potential [33]. A comprehensive resilience model assesses overall community resilience for tourism [34]. Structural Equation Modeling (SEM) has the advantage of being able to assess the overall support of residents for tourism development [35,36]. The relationships between tourism impacts, emotions, and stress are tested by the Partial Least Squares Structural Equation Modeling (PLS-SEM) [37].

This study develops the hierarchical variance analysis, a mathematical approach using Cronbach’s alpha tests, ANOVA, and linear regression analysis to analyze the socio-demographic composition of surveyed residents according to their perception on tourism impacts. Socio-demographic composition is expressed by variables such as gender, age, marital status, the condition of being native, foreign participants’ years of residence in the city, parental status, education level, participation in local associations and neighborhood groups, and the type of work in relation to tourism [7]. Analyzing socio-demographic variables supports research that recognizes existing conditions, needs, and expectations of a given population [38,39,40]. Taking into account the impacts of tourism, a combination of mathematics procedures is definitely worth additional empirical research. Pearson correlations, ANOVA analyses, and hierarchical multiple regression analyses using socio-demographic variables shows significant variance in the overall attitudes [7]. Descriptive statistics was combined with a series of independent sample t-tests to assess statistically significant differences caused by socio-demographic characteristics of residents living in a tourism destination [41]. The two-level hierarchical linear model using the fixed effects model, random intercept empty model, random coefficients model, and Cook’s distance test was used to assess the impacts of tourism conducted from perceptions of residents [42]. However, there are still few quantitative studies which mix mathematics procedures to examine how socio-demographic variables affect the residents’ perception on social, economic, and environmental impacts of tourism. The proposed hierarchical variance analysis is a vindication of our efforts to solve two main problems in the field of tourism impacts: the first is to examine the link between relevant factors and the confirmation of their significant contribution; and the second is to analyze the residents’ perception of tourism impacts according to each socio-demographic variable.

The paper is organized as follows: Section 2 introduces the mathematic procedures of the hierarchical variance analysis methodology; the results of a case analysis in a Vietnamese island destination are described in Section 3; and a conclusion and policy implication are drawn up in Section 4.

## 3. Methodology

### 3.1. Problem

The hierarchical variance analysis combines three mathematic procedures: Cronbach’s alpha tests (to assess the reliability of independent variables), the exploration of relevant factors (to measure the effect of independent variables on the perceived impacts), and a hierarchical factor confirmation (to find the one that explains most of the contribution of the relevant variables on the perceived impacts and to confirm the likely interaction). Perceived tourism impacts are expressed by dependent variables, while socio-demographic characteristics are expressed by independent variables. To process the model, dependent variables of tourism impacts *Y* were selected: Economic impacts (*Y*_1_), Socio-cultural impacts (*Y*_2_), and Environmental impacts (*Y*_3_) (Table 1). The six independent variables of socio-demographic are: Gender ($X_{1}$), Marital status ($X_{2}$), Education level ($X_{3}$), Age ($X_{4}$), Type of work ($X_{5}$), and Social network ($X_{6}$). In the first step, Cronbach’s Alpha test verifies the relevance of *X* for each tourism impact *Y*. In the next steps, an ANOVA compares the positive, negative, and overall variance of each perceived impact on tourism development. A linear regression analysis measures the effect of socio-demographic variables on three impacts confirming the result of the exploration step. By the end of the analysis, a two-factor ANOVA confirms the likely interaction between “Education level” and “Type of work” while predicting three impacts.

### 3.2. Cronbach Alpha’s Test

Cronbach Alpha’s test estimates the reliability of independent variables $X_{i}$ in each question. Suppose that we are interested in the relevance of variables $X_{i},i=\bar{1,K}$, let Z be the total test score in each question:

(1) $$Z=X_{1}+X_{2}+\ldots +X_{K}$$

The Cronbach Alpha is defined as [43]

(2) $$\alpha =\frac{K}{K-1}\left(1-\frac{\sum_{i=1}^{K}\sigma_{X_{i}}^{2}}{\sigma_{Z}^{2}}\right)$$

where $\sigma_{Z}^{2}$ is the variance of the total observed test scores, and $\sigma_{X_{i}}^{2}$ is the variance of the variable $X_{i}$.

Cronbach Alpha varies from 0 to 1. The greater value of α, the more acceptable the internal consistency among variables *X* (Table 2).

### 3.3. Exploration of Relevant Factors

A one-factor ANOVA is used to explore the relationship between an impact and a variable by measuring the effect of the socio-demographic background of the residents on the perceived economic, socio-cultural, the environmental impacts of tourism.

Suppose that the factor X consists of k treatments $T_{1},\ldots ,T_{k},$ and there are $n_{i}$ observations $Y_{i1},\ldots ,Y_{in_{i}}$ of the dependent variable Y with respect to the treatment $T_{i}\;\left(i=\bar{1,k}\right)$. The following denotations are used:

$\mu_{i.}=\frac{1}{n_{i}}\sum_{j=1}^{n_{i}}Y_{ij},$ is the treatment mean;

$E\left(Y_{ij}\right)=\frac{1}{n}\sum_{i}\sum_{j}Y_{ij}=\mu$ ($n=\sum_{j}n_{j}$), is the grand mean;

$$\alpha_{i}=\mu -\mu_{i.}$$

$\varepsilon_{ij}$: the error terms.

The impact of X on variable Y is tested using the significant difference between the k treatment means:

$H:\mu_{1}=\mu_{2}=\ldots =\mu_{k}\;\mathrm{vs}.\;K:\;\mathrm{at}\;\mathrm{least}\;\mathrm{one}\;\mathrm{mean}\;\mathrm{differs}$.

With the assumption that $\varepsilon_{ij}$ are independent normally distributed random variables with $E\left(\varepsilon_{ij}\right)=0,\;\mathrm{Var}\left(\varepsilon_{ij}\right)=\sigma^{2}$ (*), the random variables $Y_{ij}$ can be written [45] as

(3) $$Y_{ij}=\mu +\alpha_{i}+\varepsilon_{ij},i=\bar{1,k},j=\bar{1,n_{i}}$$

The deviation of $Y_{ij}$ can be separated in the variation between the treatments ($\alpha_{i}$) and within the treatments ($\varepsilon_{ij}$):

(4) $$\overset{k}{\underset{i=1}{\sum }}\overset{n_{i}}{\underset{j=1}{\sum }}{\left(y_{ij}-\mu \right)}^{2}=\overset{k}{\underset{i=1}{\sum }}n_{i}{\left(\mu_{i.}-\mu \right)}^{2}+\overset{k}{\underset{i=1}{\sum }}\overset{n_{i}}{\underset{j=1}{\sum }}{\left(y_{ij}-\mu_{i.}\right)}^{2}$$

or

SSTotal (total sum of square)=SST (sum of squared treatment)+SSE (sum of squared errors)

The difference among the treatment means is tested as

$$H:\alpha_{i}=0\left(\forall i\right);K:\;\mathrm{At}\;\mathrm{least}\;\mathrm{one}\;\alpha \neq 0$$

Results from comparing SST with SSE, if the weight of SST is equal or less than that of SSE. There should be no difference among k treatments, otherwise, there is a significant disparity among the k means, which results from an impact of factor F on the dependent variable Y.

Because the values of $\mu \;\mathrm{and}\;\mu_{i}$ are unknown, they are replaced by an estimation of $\bar{y}$. (sample grand mean) and ${\bar{y}}_{i}$ (sample treatment mean):

(5) $$\overset{k}{\underset{i=1}{\sum }}\overset{n_{i}}{\underset{j=1}{\sum }}{\left(y_{ij}-\bar{y}\right)}^{2}=\overset{k}{\underset{i=1}{\sum }}n_{i}{\left({\bar{y}}_{i.}-\bar{y}\right)}^{2}+\overset{k}{\underset{i=1}{\sum }}\overset{n_{i}}{\underset{j=1}{\sum }}{\left(y_{ij}-{\bar{y}}_{i.}\right)}^{2}$$

By dividing SST and SSE by their corresponding degrees of freedom ($\nu_{1}=k-1$ and $\nu_{2}=n-k$) to obtain MST (mean square of treatment) and MSE (mean square of error), the sampling distribution of the ratio F=MST/MSE is a Fisher distribution with $\nu_{1}\;\mathrm{and}\;\nu_{2}$ degrees of freedom. If $F>F_{\alpha }$, the hypothesis H is rejected.

### 3.4. Hierarchical Factor Confirmation

#### 3.4.1. Linear Regression Analysis

The multiple linear regression is

$$Y=\beta_{0}+\beta_{1}X_{1}+\beta_{2}X_{2}+\ldots +\beta_{p}X_{p}+\varepsilon$$

where ε is the random error, following the normal distribution with $E\varepsilon =0,\mathrm{Var}\varepsilon =\sigma^{2}$.

The following linear regression equation is estimated:

$$EY=\beta_{0}+\beta_{1}X_{1}+\beta_{2}X_{2}+\ldots +\beta_{p}X_{p}$$

Using either the least square method or the maximum likelihood estimation, one finds the estimators of the p coefficient $\beta_{i}$ as $b_{i}$, following the estimated linear regression equation:

$$\hat{Y}=b_{0}+b_{1}X_{1}+\ldots +b_{p}X_{p}$$

To further explore the bilateral relationship between a variable and an impact, a one-way ANOVA is used. Independent variables will be combined to find out about their simultaneous effect on the outcome. As a result, several hierarchical models are considered to find the one that explains most of the contribution of the relevant variables on the impacts.

Suppose that *p* independent variables affect variable Y, p models are used with 1, 2, ..., p predictors with respect to the largest ${\hat{R}}^{2}$ (adjusted $R^{2}$). Particularly, among p. linear models containing 1 predictor, i.e., $\hat{Y}=b_{0}+b_{i}X_{i}$, the one providing the largest ${\hat{R}}^{2}$ will be selected; among $\frac{p\left(p-1\right)}{2}$ models containing two predictors, i.e., $\hat{Y}=b_{0}+b_{i_{1}}X_{i_{1}}+b_{i_{2}}X_{i_{2}}$, the one with the largest ${\hat{R}}^{2}$ will be plotted, until the last one containing all p variables are used. After that, the model providing the largest ${\hat{R}}^{2}$ is selected.

Suppose the dataset consists of n sets ${\left\{\left(y_{i},x_{1i},\ldots ,x_{pi}\right)\right\}}_{i=\bar{1,n}}$. Using a similar option as the one in Equation (5), the total sum of squared difference consists of two sources [40]:

(6) $$\overset{n}{\underset{i=1}{\sum }}{\left(y_{i}-\bar{y}\right)}^{2}=\overset{n}{\underset{i=1}{\sum }}{\left(y_{i}-\hat{y_{i}}\right)}^{2}+\overset{n}{\underset{i=1}{\sum }}{\left(\hat{y_{i}}-\bar{y}\right)}^{2}$$

where $\bar{y}$ is the sample grand mean of Y, and $\hat{y_{i}}$. are the estimated values of Y given the i^th^ value of X.

Denoting $SSTotal=\sum_{i=1}^{n}{\left(y_{i}-\bar{y}\right)}^{2},SSE=\sum_{i=1}^{n}{\left(y_{i}-\hat{y_{i}}\right)}^{2},SSR=\sum_{i=1}^{n}{\left(\hat{y_{i}}-\bar{y}\right)}^{2}$, Equation (6) can be rewritten as SSTotal=SSE+SSR. SSE measures the lack of fit of the regression model, and SSR measures the variation that can be explained by the regression model. The determination coefficient of $R^{2}$ is defined by the ratio SSR/SSE; the larger $R^{2}$ is, the better the model fits the data. However, when increasing the number of variables, $R^{2}$ also increases; therefore, it is inappropriate to use this number to assess how well the model fits the data. The adjusted $R^{2}$ is introduced to deal with this problem:

(7) $$R_{adj}^{2}=1-\frac{\frac{SSE}{df_{e}}}{\frac{SSTotal}{df_{t}}}=1-\left(1-R^{2}\right)\times \frac{n-1}{n-p-1}$$

Taking into account the degrees of freedom ($df_{e}=n-p-1,df_{t}=n-1$), the adjusted $R^{2}$ increases when the increase in $R^{2}$ is more than one would expect to see by chance.

Remarkably, the Fisher test is performed in a similar way to explore the relevant steps. The following test problem is considered:

$H:\beta_{1}=\ldots =\beta_{l}=0$ (reduced model);
$K:\;\mathrm{at}\;\mathrm{least}\;\mathrm{one}\;\beta_{i}\;\mathrm{differs}\;\left(\mathrm{full}\;\mathrm{model}\right)$.

Taking into account the Fisher test, the error between the estimators of coefficient and the observed values is evaluated [46]: the sum of the squared error of the two models: SSE(R) and SSE(F) (the reduced and the full one, respectively). While the estimator of $y_{i}$ is ${\hat{y}}_{i}$ in the full model, deducing $\left(F\right)=\sum_{i=1}^{n}{\left(y_{i}-{\hat{y}}_{i}\right)}^{2}$, the estimator in reduced model is $\bar{y}\;\left(\forall i\right)$, deducing $SSE\left(R\right)=\sum_{i=1}^{n}{\left(y_{i}-\bar{y}\right)}^{2}$. Because of Equation (6), SSE(R)≥SSE(F). The following cases are possible:

- In case SSE(F) is close to SSE(R), the full model does not reduce the total variance of SST and SSR (the variation explained by the regression model) is limited. The reduced model is selected;
- On the contrary, in case SSE(F) differs significantly from SSE(R), the full model reduces substantially and the total variance and the full model is selected.

The ratio (F) measures the difference between SSE(F) and SSE(R):

(8) $$F=\frac{SSE\left(R\right)-SSE\left(F\right)}{df_{r}-df_{f}}:\frac{SSE\left(F\right)}{df_{f}}$$

In the case that the full model contains p variables, the degrees of freedom of SSE(F) is given as n−p−1, and of SSE(R) it is given as n−1. Take notice that in Equation (6) SSE(R)−SSE(F)=SSTotal−SSE(F)=SSR(F) can be rewritten as

(9) $$F=\frac{SSR}{p}:\frac{SSE\left(F\right)}{n-p-1}=\frac{MST}{MSE}$$

If $F>F_{\alpha },$ which means the difference is significant, H is rejected and the full model is used for the prediction.

The Fisher test is used to compare the two models and allows the choice of the one with the smallest variance.

#### 3.4.2. Two-Factor ANOVA

Supposing one wants to see the effect of factor A containing a levels and factor B containing b levels on the outcome variable Y, the problem can be formulated using the following symbols [47]:

$y_{ijk}$ are the observations to the i^th^ level of factor A and j^th^ level in factor B where $k=\bar{1,n_{ij}},\sum_{i,j}n_{ij}=n$ (but $n_{ij}$ are commonly assumed to be the same as n/ab);

$\mu =EY=\frac{1}{n}\sum_{i,i,k}y_{ijk}$ is grand mean;

$\mu_{ij.}=\frac{1}{n_{ij}}\sum_{k}y_{ijk}$ is the mean at the i^th^ level in factor A and j^th^ level in factor B;

$\mu_{i..}=\frac{1}{b.\;n_{ij}}\sum_{j,k}y_{ijk}$ is the mean at the i^th^ level of factor A, $\mu_{.j.}=\frac{1}{a.n_{ij}}\sum_{i,k}y_{ijk}$ is the mean of the j^th^ level in factor B;

$\alpha_{i}=\mu -\mu_{i..}$ is the main effect of factor A, $\beta_{j}=\mu -\mu_{.j.}$ is the main effect of factor B;

$\varepsilon_{ijk}$ are the random error variable satisfying $E\left(\varepsilon_{ijk}\right)=0,\;Var\left(\varepsilon_{ijk}\right)=\sigma^{2}$.

Along the same idea as the one-factor ANOVA, the two-factor ANOVA can be written as

(10) $$y_{ijk}=\mu +\alpha_{i}+\beta_{j}+{\left(\alpha \beta \right)}_{ij}+\varepsilon_{ijk}$$

where ${\left(\alpha \beta \right)}_{ij}$ is the effect of the interation between factor A and B. As a result, the total variance can be partitioned as

(11) $$\underset{i,j,k}{\sum }{\left(y_{ijk}-\mu \right)}^{2}=\underset{i,j,k}{\sum }{\left(y_{ijk}-\mu_{ij.}\right)}^{2}+\underset{i,j}{\sum }n_{ij}{\left(\mu_{ij.}-\mu \right)}^{2}$$

of notice $SSE=\sum_{i,j,k}{\left(y_{ijk}-\mu_{ij.}\right)}^{2}$ and

(12) $$\underset{i,j}{\sum }n_{ij}{\left(\mu_{ij.}-\mu \right)}^{2}=\underset{i}{\sum }b.n_{ij}{\left(\mu_{i..}-\mu \right)}^{2}+\underset{j}{\sum }a.n_{ij}{\left(\mu_{.j.}-\mu \right)}^{2}+\underset{i,j}{\sum }n_{ij}{\left(\mu_{ij.}-\mu_{i..}-\mu_{.j.}+\mu \right)}^{2}$$

Therefore, $\sum_{i}b.n_{ij}{\left(\mu_{i..}-\mu \right)}^{2}=SSA,\sum_{j}a.n_{ij}{\left(\mu_{.j.}-\mu \right)}^{2}=SSB,\sum_{i,j}n_{ij}{\left(\mu_{ij.}-\mu_{i..}-\mu_{.j.}+\mu \right)}^{2}=SS\left(AB\right),$ then

(13) SS Total=SSA+SSB+SS(AB)+SSE

This means the total sum of square of variance can be partitioned into one source from factor A, one from factor B, one from their interaction, and one from the random error. Replacing the estimators for the unknown parameters in this model, Equation (14) can be written as

(14) $$\underset{i,j,k}{\sum }{\left(y_{ijk}-\bar{y}\right)}^{2}=\underset{i}{\sum }b.n_{ij}{\left({\bar{y}}_{i..}-\bar{y}\right)}^{2}+\underset{j}{\sum }a.n_{ij}{\left({\bar{y}}_{.j.}-\bar{y}\right)}^{2}+\underset{i,j}{\sum }n_{ij}{\left({\bar{y}}_{ij.}-{\bar{y}}_{i..}-{\bar{y}}_{.j.}+\bar{y}\right)}^{2}+\underset{i,j,k}{\sum }{\left(y_{ijk}-{\bar{y}}_{ij.}\right)}^{2}=SSA+SSB+SS\left(AB\right)+SSE$$

At this point, the Fisher test assesses these sources of variance:

*Problem 1*: test the hypothesis: H: $\mu_{1..}=\mu_{2..}=\ldots =\mu_{a..}$ vs. K: at least one mean differs. The Fisher statistic is $F_{A}=\frac{SSA/df_{a}}{SSE/df_{e}}=\frac{MSA}{MSE},df_{a}=a-1$;

*Problem 2*: test the hypothesis: H: $\mu_{.1.}=\mu_{.2.}=\ldots =\mu_{.b.}$ vs. K: at least one mean differs. The Fisher statistic is written as $F_{B}=\frac{SSB/df_{b}}{SSE/df_{e}}=\frac{MSB}{MSE},\;df_{b}=b-1$;

*Problem 3*: test the hypothesis: H: there is no interaction between both factors and K: The Fisher statistic is written as $F_{AB}=\frac{\frac{SS\left(AB\right)}{df_{ab}}}{\frac{SSE}{df_{e}}},df_{ab}=\left(a-1\right)\left(b-1\right),df_{e}=df_{t}-df_{a}-df_{b}-df_{ab}$.

This complex process is performed using the R software, in which the *p*-value allows one to decide about the H. If the *p*-value is smaller than the critical value α, H is rejected.

## 4. The Case Analysis

### 4.1. The Ly Son Destination

**Figure 1:**
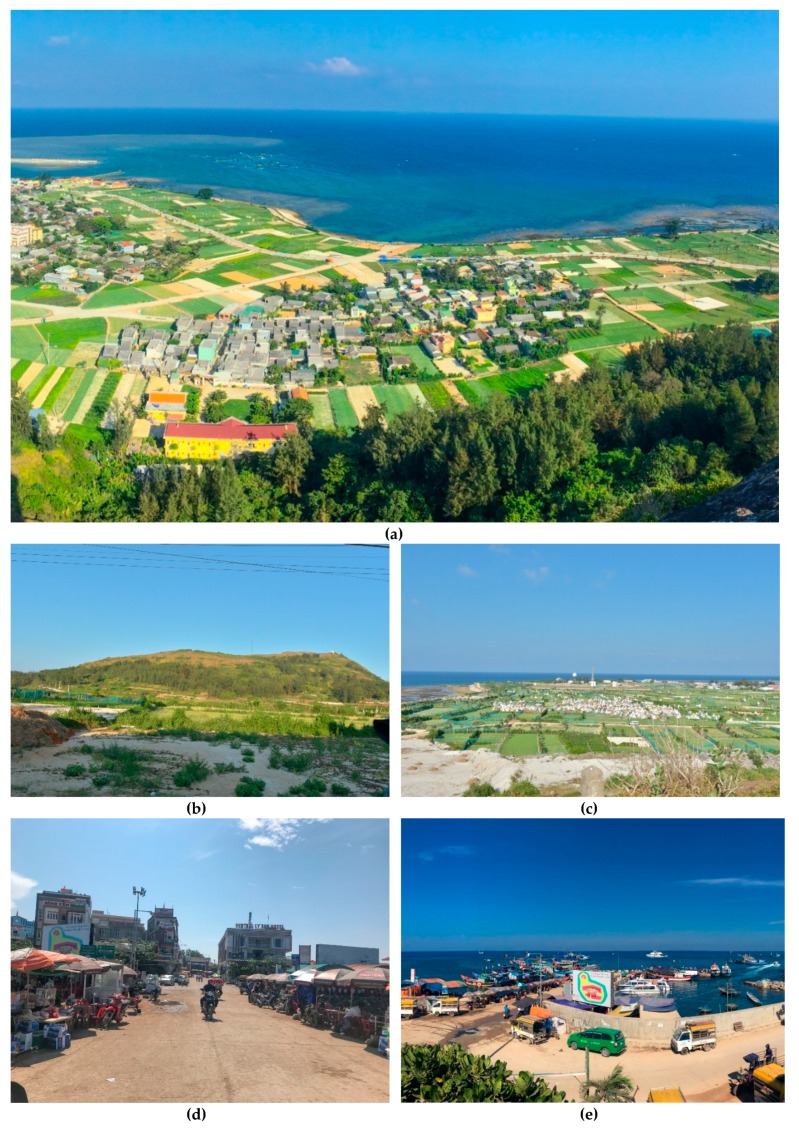
The total landscape and tourism areas on Ly Son Island (photo by authors, 2018). (**a**) Overview of Ly Son landscape; (**b**) mountainous forests; (**c**) farming tourism areas; (**d**) residential tourism recreations (R); (**e**) the sandy coasts of the island (C).

Ly Son Island is the most attractive destination in Quang Ngai province, on the South Central Coast, Vietnam. Ly Son has substantial biodiversity on the land and in the sea. The biodiversity is well protected in the Ly Son Marine Protected Areas. The island can be categorized into four main areas: mountainous forest, farms, residential areas, and the coast (Figure 1). The most attractive tourist sites are the resorts in Hang Cau, Bac An Hai, and Nam An Vinh; other spots include the Sau volcanic cave, the garlic fields, and the beaches of Chua Duc, Bac An Hai, and Hang Cau. The island attracted about 95,000 visitors in 2015, and over 230,000 visitors in 2018. Tourism development contributes significantly to the local economy: the tourism revenue is estimated at USD 12 million in 2018. Some of the negative consequences of rapid tourism development on the island can be seen to have affected the master planning, fishing, social life, and environment. Massive motels and hotels have broken with the master plan and the overview of the total landscape. Near-shore seafood has been exhausted due to over-fishing. A number of shops and spontaneous tourist stalls have sprung up around the monuments and natural landscapes, making the island appear unsightly. The overbalance of tourists on the island at the weekend influences the normal life of residents. Environmental pollution has become a problem due to the increase in water use, waste, and sewage [48].

### 4.2. Data Collection

This study aimed to collect information on the socio-demographic background of residents and survey their perception of tourism impacts. The questionnaire was divided into two main parts: the first part entailed socio-demographic questions; the second part was about perceptions of the economic, socio-cultural, and environmental impacts of tourism. Finally, respondents were asked about their social network in yes/no questions. The socio-demographic information dealt with gender, marital status, education level, age, type of work, and social network. Marital status included two categories: “married” and “single”. Education levels were classified into four groups: “Primary education or below”, “Secondary school”, “High school” and “Beyond high-school level”. Four age groups were inventoried: “18–25 years”, “26–42 years”, “43–55 years” and “Older than 55 years”. The type of work question included as alternatives: “Farmer”, “Fishermen”, “Trade and Tourism service”, and “Free labor and Other”. The questionnaires were completed during a field trip in September 2018. The sample includes 452 residents selected according to a stratified random sample design.

The perception scale used items on economic, socio-cultural, and environmental impacts, and an overall assessment. The items were quantified using a 5-point Likert scale, which expressed ‘strong disagreement’ as (1), ‘disagreement’ (2), ‘neutral’ (3), ‘agreement’ (4), and ‘strong agreement’ (5).

### 4.3. Descriptive Statistics

The Cronbach Alpha of the economic impacts group is 0.811, of the socio-cultural impacts group is 0.785, and of the environmental impacts group is 0.823. The results show that the questions in each group were relevant, and consequently, all questions could be used in the analysis.

#### 4.3.1. Socio-Demographic Variables

Gender ($X_{1}$): almost two thirds of surveyed respondents were male.

Marital status ($X_{2}$): a majority of respondents (94.5%) were married.

Education levels ($X_{3}$): there were equal percentages among the surveyed residents’ education levels: primary or below, secondary, high school or beyond; while the number of cases holding high school level and beyond high-school level were almost the same.

Age ($X_{4}$): over half of the respondents were over 43 years old, while young laborers (between 18 and 25 years old) accounted for 2%.

Types of work ($X_{5}$): almost half of residents were farmers, while about 14% of them went fishing for living; about 18% of cases worked in trade and tourism services, and a small proportion of residents were officials.

Social participation ($X_{6}$): a third of respondents admitted to participating in social activities in the community.

#### 4.3.2. Tourism Impacts

The scale means and standard deviations were calculated in the positive and negative assessment on three impacts, and the mean of overall assessment was considered (Table 3).

#### 4.3.3. Correlations between Socio-Demographic Variables and Tourism Impacts

On the basis of the correlation coefficients, residents had a clear assessment of the negative economic impacts (with more pessimism among single, higher educated, younger, and free labor residents), and overall environmental satisfaction (with more pessimism among male, single, higher educated, farming–fishing, and non-participating residents) (Table 4).

### 4.4. Socio-Demographic Effects on Attitudes and Tourism

#### 4.4.1. Effects of gender

No marked effect of gender on economic impacts was found. Male residents were more optimistic than the woman in terms of positive socio-cultural impacts (F=3.79, p=0.052). Men were more worried than the women about negative environmental impacts (F=12.35,p<0.001) and less satisfied in terms of the overall environmental impacts (F=8.87,p<0.01).

#### 4.4.2. Effects of Marital Status

Economic impacts: married residents were more satisfied than singles ones about the positive impacts (F=4.32,p<0.05), less worried about negative impacts (F=10.65,p<0.01), and more optimistic in their overall assessment (F=5.43,p<0.05).

Socio-cultural impacts: married residents were more satisfied in their overall assessment than the single ones (F=5.0,p<0.05).

Environmental impacts: married residents were more satisfied than single ones on the positive impacts (F=12.7,p<0.001), and on the overall assessment (F=8.13,p<0.01).

#### 4.4.3. Effect of Education Levels

Economic impacts: education levels had the main effects on negative economic impacts. Residents educated to a higher level worried more about the negative impacts than others (F=4.59,p<0.01). Residents who studied to a high-school level worried most, and those with a primary level or below worry least

Socio-cultural impacts: education levels clearly affected negative socio-cultural impacts (F=4.07,p<0.01). Residents educated to a higher level worried more the ones educated to a lesser level, while the assessments of the others were not clear

Environmental impacts: education levels affected the positive aspects most (F=7.17,p<0.001). The negative aspects (F=11.26,p<0.001) and the overall assessment (F=14.45,p<0.001) of the environmental impacts were less affected. Residents with primary level of education or below = and secondary school levels were optimistic about the positive assessments, while those with higher school educations showed more pessimism. Regarding the negative assessment, the higher the education level of the residents, the less they worry, except for those with a level above high school (who’s assessment was unclear). Overall, the satisfaction of residents increased along with their education level regardless of the ideas from the group with the highest level of education.

#### 4.4.4. Effects of Age

Economic impacts: age had significant effects on positive economic expectations from tourism (F=3.64,p<0.05). The youngest residents expressed less satisfaction while those between 26 and 42 year had the most positive expectations. The ideas of seniors were not as clear as those of the other age groups, although they were less satisfied than the age group of 26–42 (t=2.24,p<0.05).

Socio-cultural impacts: the main effects of age on the negative and overall assessment were found (F=6.76,p<0.001 and F=5.82,p<0.001, respectively). T-tests confirmed this effect: the most pronounced negative assessments were found for the age groups 18–25 and 43–55 (t=2.14,p<0.05), group 26–42 and 43–55 (t=4.14,p<0.001), group 26–42 and 55–81 (t=2.8,p<0.01).

Environmental impacts: a significant effect of age on the positive assessment (F=6.42,p<0.001) and overall assessment (F=4.52,p<0.01) were found. The t-test results include in following: 26–42 vs. 18–25 (t=1.90,p<0.05), 43–55 vs. 18–25 (t=2.07,p<0.05), over 55 vs. 18–25 (t=1.96,p<0.05). They show that the youngest residents expected the least positive aspects of environmental impacts, while residents between 43 and 54 years expected the most. No clear differences were found in terms of negative aspects. The overall satisfaction followed the same trend: the youngest group was less satisfied than the middle-aged group.

#### 4.4.5. Effects of type of work

Economic impacts: a significant effect of the type of work on both the positive and negative aspects of the economic impacts was found (F=2.46,p<0.05 and F=5.82,p<0.001, respectively). T-tests indicated that residents working in trade and tourism services had the most positive expectation: farmer vs. trade and tourism (t=−2.37,p<0.01), fishermen vs. trade and tourism (t=−2.26,p<0.05), free labor vs. trade and tourism (t=−2.56,p<0.01). Free laborers demonstrated the most pessimism on the negative impacts: farmer vs. free labor (t=−4.13,p<0.001), fishermen vs. free labor (t=−5.1,p<0.001), trade and tourism vs. free labor (t=−4.22,p<0.001).

Socio-cultural impacts: types of work influenced negative effects (F=3.22,p<0.05). The t-test results showed that farmers are the most worried about the negative aspects of socio-cultural impacts: trade and tourism vs. farmer (t=−2.01,p<0.05), fishermen vs. farmer (t=−1.78,p<0.05), officials vs. farmer (t=−1.34,p<0.1).

Environmental impacts: significant effects on the positive, negative, and overall assessment were found (F=2.95, p<0.05;F=4,p<0.01 and F=4.43,p<0.01, respectively). Results of the ANOVA and t-tests showed that farmers were the least satisfied in terms of the positive and overall assessment on environmental impacts; free labors worried least about the negative impacts and were most satisfied on the overall assessment.

#### 4.4.6. Effects of Social Networks

Economic impacts: the ANOVA result showed a significant difference in the overall assessment (F=5.58,p<0.05): residents who participated in social organizations felt more satisfied.

Socio-cultural impacts: no effect was found.

Environmental impacts: significant effects on the negative and overall assessment were found (F=10.17,p<0.01 and F=55.34,p<0.001, respectively). Residents with a social network worried more and were less satisfied than those without a social network.

### 4.5. Socio-Demographic Effects Analyzed with a Linear Regression Model

The socio-demographic variables were put into the linear regression analysis to analyze their simultaneous effects on the perceptions of tourism development.

#### 4.5.1. Economic Impacts

Table 5 shows the significant effects of some socio-demographic variables on the negative sides and overall assessment of the economic impacts. In particular, marital status, education level, type of work, and social network contributed 7.91% to the variability of negative impacts explained by the model. Single, higher educated, and socially participating residents felt more pessimistic about these negative impacts. Marital status, education level, type of work, and social network accounted for 2.7% of the variability of the overall assessment score explained by the model. This shows that married, socially participating residents demonstrated a higher overall satisfaction in terms of economic impacts.

#### 4.5.2. Socio-Cultural Impacts

Male and socially participating residents felt more optimistic about the positive aspects of tourism which contribute 2.24% to the variability of the positive scores explained by the model. Regarding the negative aspects, higher educated residents worried more than fishermen, official agents, and those working in trade and tourism. This explains the significant variance of 7.5% of the negative scores. Overall, married and socially participating residents were more satisfied in terms of the socio-cultural impacts of tourism development (Table 6).

#### 4.5.3. Environmental Impacts

Socio-demographic variables explain the significant variances of the assessment scores on environmental impacts. Marital status, education level, and type of work explain 7.82% of the variability of positive scores; gender, education level, type of work, and social network explain 14.98% of the variability of negative scores; gender, marital status, education level, type of work, and social network explain 17.79% of the variability of overall scores. Married, lower educated residents, and free laborers felt more satisfied in terms of the positive aspects; female, secondary- and high-school educated, free laborers, and others who did not participate in social activities worried less about the negative aspects. In the overall assessment, female, married people, free laborers, and nonsocially participating residents felt more optimistic about the environmental impacts of tourism development (Table 7 and Table 8).

### 4.6. Interaction Effects on Three Tourism Impacts Analyzed Using a Two-Factor ANOVA

**Figure 2:**
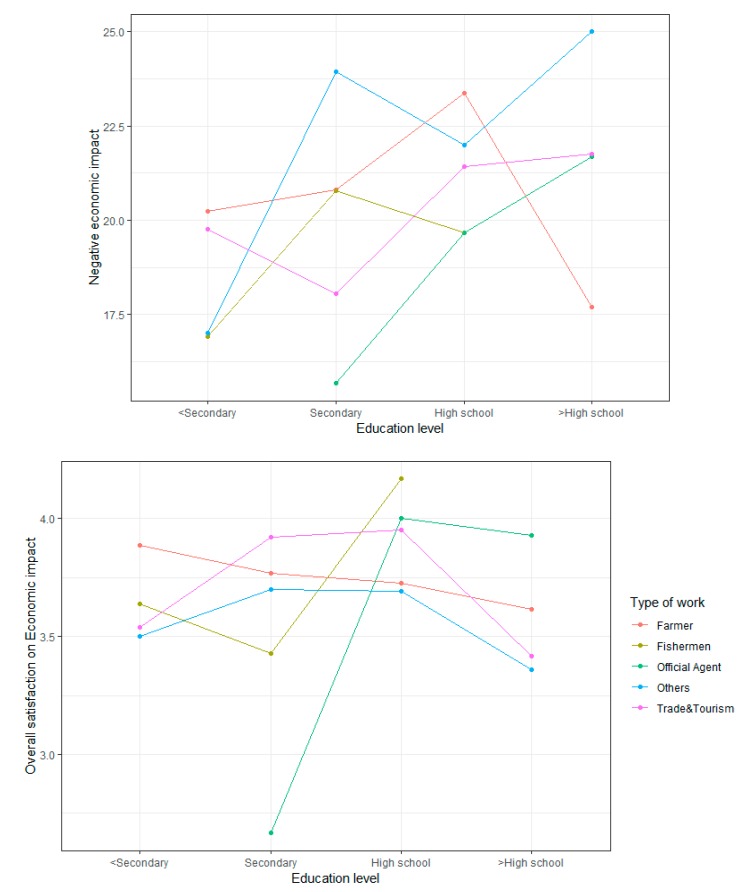
Effect of the interaction between education level and type of work on the economic impacts.

**Figure 3:**
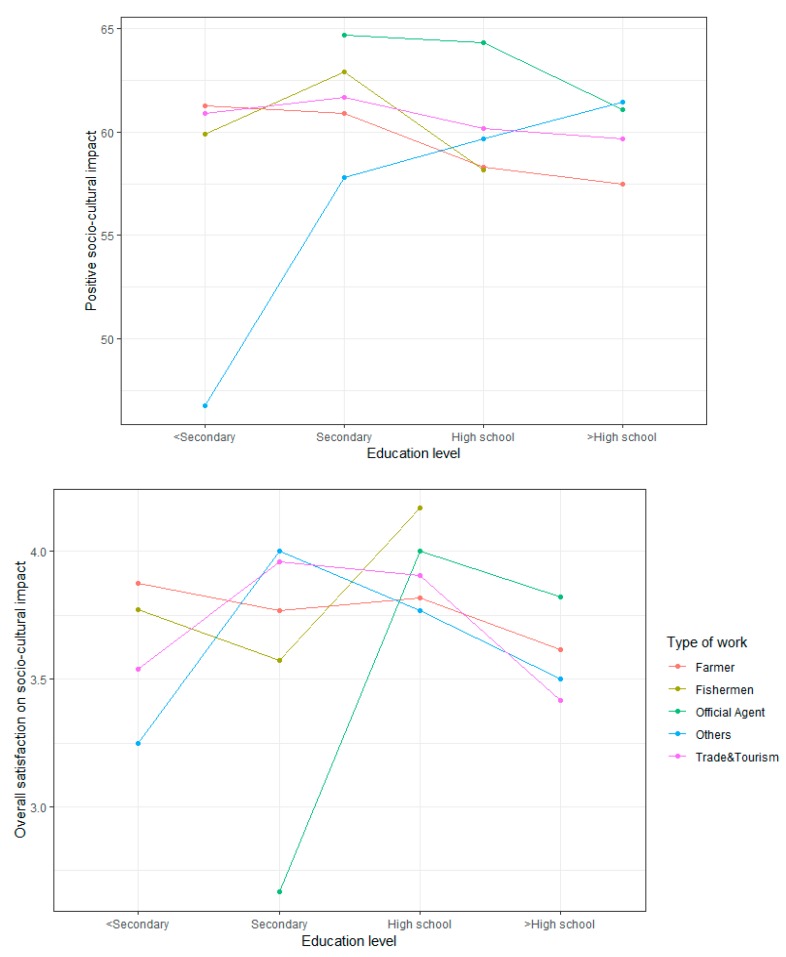
Effect of interaction between “Education level” and “Type of work” on the socio-cultural impacts.

**Figure 4:**
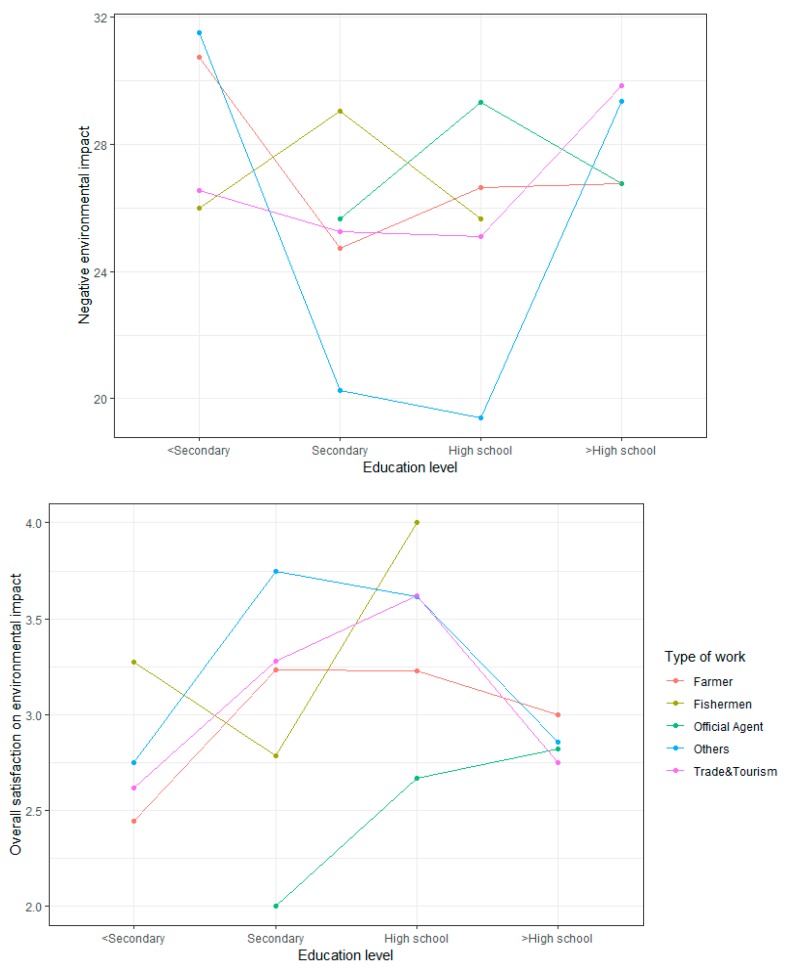
Effect of interaction between “Education level” and “Type of work” on the environmental impacts.

Types of work and education levels are two important factors in predicting the satisfaction of the residents. The two-factor ANOVA confirmed the interaction plots of the tourism attitude dimensions on education levels by different types of work, controlling the effects of the variables related to tourism attitudes (i.e., gender, marital status, age, social network). The two-factor ANOVA results show a significant interaction for “Education level” × ”Type of work” and on negative and overall economic scores (Figure 2), on positive and overall socio-cultural scores (Figure 3), and on negative and overall environmental scores (Figure 4). The effects of “Education level” on tourism impacts are moderated by “Type of work”.

Significant effects of the interaction between “Education level” and “Type of work” exist in terms of the negative economic impacts and overall assessment. For the negative economic impact, dramatic changes were observed between the group of “Beyond high-school level” and “Primary level or below”; free laborers with primary level or below had the smallest negative scores, while those with a level above high school showed most pessimism. Farmers with a primary level of education or below showed the largest negative scores; on the contrary, those with the highest level of education demonstrated the smallest. On the overall assessment, fishermen and officials with secondary level of education felt the least satisfied, but those with a high-school level of education felt most satisfied compared with the two others. Adding the interaction increases the adjusted R-squared to 10.76% in the negative model (nearly 3% more than without interaction) and by 4.6% in the overall model (1% higher than without interaction).

The interaction between “Education level” and “Type of work” is a significant predictor of the positive aspects and overall socio-cultural impact. Free laborers with primary level of education or below felt least pessimistic, while those with above a high-school level worried the most in terms of the socio-cultural impacts of tourism. Farmers with a primary level of education or below ranked first in scores but those with highest educated level worried least. Officials worried more than other groups. In the overall socio-cultural satisfaction, a difference is seen between the “Secondary” group and the “High school” group: officials and fishermen with a secondary level of education were the two least satisfied groups, while those with a high-school level became the two most satisfied groups out of the five. The interaction increases the adjusted R-squared in the positive model by 3.44% and confirms the additive effect in the negative model.

A two-factor ANOVA showed the significant contribution of the interaction between “Education level” and “Type of work” on the environmental impact. Regarding negative environmental scores, for residents with primary level of education or below, farmers ranked first, free labors ranked second, and trade and tourism services were the least. This comparison changes a lot for residents who obtained a higher education level: farmers worried the least (together with officials), those belonging to trade and tourism services worried most, followed by free laborers. Regarding overall environmental scores, a difference was observed between the primary education or below group and the higher education level: farmers in the first group felt least satisfied but most satisfied in the second one; those working in the trade and tourism service ranked third for satisfaction but fell down to the last. The interaction and these two factors explain 13.58% of the total variance in the negative model and 14.2% in the overall model, which is a substantial contribution to the whole model.

## 5. Discussion

Hierarchical variance analysis is a combination mathematical procedures that allows one to assess the perceived tourism impacts. It provides quantitative procedures to analyze the variance hierarchically, as well as explaining it using the factors or the regression model in the total variance, making it feasible and straightforward to apply. The approach entails two steps: (i) exploration of the relevant factors; and (ii) confirmation of their significant contribution and the effect of their interaction in explaining the residents’ assessment. The model is comprehensive and requires uncomplicated calculation compared with other mathematics models such as the Exploratory Factor Analysis (EFA) and Confirmation Factor Analysis (CFA). Hence, it is applicable to the data collected by structured interviews. However, this approach has some limitations. When the questionnaire is not well designed, the independence between factors is violated. As a result, the regression model does not follow the additive rule. In other situations, the Fisher test does not produce a significant result, which means the linear regression model is invalid. Under these conditions, one has to apply other mathematical models such as a Structural Equation Model (SEM), a Bayesian network, or others.

The study’s findings show that at the core of solving the negative impacts of tourism development is the promotion of sustainable tourism development. Particularly Vietnam, the findings suggest significant solutions for small islands, with their relative limited surface and their relatively limited natural resources. Firstly, tourism should be managed in an interdisciplinary manner. The government should plan to raise awareness of the noneconomic aspects of tourism development among the public, including environmental and socio-culture aspects. While tourism revenue continues be a most important priority, which can be improved by an increase in visitors and the development of tourism infrastructures, the control of natural resource degradation, environmental pollution, and some negative changes in the socio-cultural life of residents should be taken into account in tourism management and strategic tourism planning at both provincial and local levels. Secondly, community participation (CP) in tourism planning and development should be brought to the forefront. A socio-demographic survey provides the input data for the perception analysis of the local residents’ support for tourism development, and their local participation in tourism planning. The findings provide a better understanding of residents’ perceptions of the local economy, and the perceived impacts of tourism development on society, the economy, and environment. This offers a scientific basis to help deal with problems emerging during tourism development and also promote the participation of locals in tourism planning and development.

## 6. Conclusions

This study investigates the effects of socio-demographic variables on residents’ perception towards tourism development in Ly Son Island, Vietnam: the bilateral and simultaneous relationships were assessed using a one-factor ANOVA to explore the relationships and then a linear regression analysis to confirm them. Furthermore, the interaction between two important factors (“Education level” and “Type of work”) is also explored by a hierarchical confirmation.

The results show that no marked effect of gender on the impacts of tourism is found, while farmers, younger, higher educated, and socially participating residents have negative assessments in terms of tourism impacts. Married and socially participating residents demonstrate higher overall satisfaction in terms of the economic impacts, and are more satisfied on the socio-cultural impacts of tourism development. Female, married people, free laborers, and nonsocially participating residents feel more optimistic about the environmental impacts of tourism development. The interaction between “Education level” and “Type of work” contributes significantly to the economic, socio-cultural, environmental impacts, and the overall impact.

## Figures and Tables

**Table 1:** Dependent variables of tourism impacts.

$\mathrm{Economic}\;\mathrm{Impacts}\;\left(Y_{1}\right)$			$\mathrm{Socio}-\mathrm{Cultural}\;\mathrm{Impacts}\;\left(Y_{2}\right)$			$\mathrm{Environmental}\;\mathrm{Impacts}\;\left(Y_{3}\right)$		
$Y_{1}^{+}$	$Y_{1}^{-}$	$Y_{1}$	$Y_{2}^{+}$	$Y_{2}^{-}$	$Y_{2}$	$Y_{3}^{+}$	$Y_{3}^{-}$	$Y_{3}$
Positive	Negative	Overall	Positive	Negative	Overall	Positive	Negative	Overall

**Table 2:** The common usage of Cronbach Alpha [44].

Cronbach Alpha	Internal Consistency
0.9≤α	Excellent
0.8≤α<0.9	Good
0.65≤α<0.8	Acceptable
0.5≤α<0.65	Poor
α<0.5	Unacceptable

**Table 3:** The (scale) mean and standard deviation of the assessment on three impacts.

	$\mathrm{Economic}\;\mathrm{Impacts}\;\left(Y_{1}\right)$			$\mathrm{Socio}-\mathrm{Cultural}\;\mathrm{Impacts}\;\left(Y_{2}\right)$			$\mathrm{Environmental}\;\mathrm{Impacts}\;\left(Y_{3}\right)$		
	$Y_{1}^{+}$	$Y_{1}^{-}$	$Y_{1}$	$Y_{2}^{+}$	$Y_{2}^{-}$	$Y_{2}$	$Y_{3}^{+}$	$Y_{3}^{-}$	$Y_{3}$
(Scale) mean	3.7	3.5	3.8	3.8	2.8	3.8	3.8	3.1	3.1
Standard deviation	0.603	0.808	0.665	0.430	0.458	0.667	0.508	0.835	1.007

**Table 4:** Correlations between socio-demographic variables and tourism impacts.

	Economic Impact			Socio-Cultural Impact			Environmental Impact		
	$Y_{1}^{+}$	$Y_{1}^{-}$	$Y_{1}$	$Y_{2}^{+}$	$Y_{2}^{-}$	$Y_{2}$	$Y_{3}^{+}$	$Y_{3}^{-}$	$Y_{3}$
$X_{1}$	−0.062	0.052	0.026	−0.097 (.)	−0.007	−0.022	0.003	−0.172 (***)	0.147 (**)
$X_{2}$	−0.058	0.159 (**)	−0.078	−0.004	0.079	−0.078	−0.117 (*)	0.042	−0.117 (*)
$X_{3}$	0.03	0.15 (**)	−0.032	−0.021	0.119 (*)	−0.05	−0.088 (.)	−0.103 (*)	0.133 (**)
$X_{4}$	−0.067	−0.118 (*)	0.037	0.014	−0.156 (**)	0.063	0.088 (.)	0.058	−0.053
$X_{5}$	0.047	0.115 (*)	−0.038	−0.047	−0.034	−0.002	0.092 (.)	−0.19 (***)	0.178 (***)
$X_{6}$	0.026	−0.04	−0.11 (*)	−0.087 (.)	0.011	−0.086 (.)	0.063	−0.148 (**)	0.33 (***)

Note: (.) p<0.10; (*) p<0.05; (**) p<0.01; (***) p<0.001.

**Table 5:** Linear regression analysis for economic impacts and socio-demographic variables.

Impact	Predictors	β	$SE_{\beta }$	$R^{2}$	$\mathrm{Adjusted}\;R^{2}$	F Test Value
$Y_{1}^{+}$	$X_{1}$-Female	−0.637	0.402	0.052	0.0273	2.102 (*)
	$X_{2}$-Single	−1.245	0.888			
	$X_{3}$-Secondary	1.142 (*)	0.479			
	$X_{5}$-Trade and Tourism	0.982 (.)	0. 55			
	$X_{6}$-Not participate	−0.449	0.42			
$Y_{1}^{-}$	$X_{2}$-Single	2.176 (.)	1.172	0.1	0.0791	4.749 (***)
	$X_{3}$-Secondary	1.446 (*)	0.631			
	$X_{3}$-High school	2.731 (***)	0.809			
	$X_{3}$-Beyond high school	2.019 (*)	0.946			
	$X_{5}$-Others	1.949 (*)	0.891			
	$X_{6}$-Not participate	−1.399 (*)	0.553			
$Y_{1}$	$X_{2}$-Single	−0.465 (**)	0.16	0.0575	0.0355	2.605 (**)
	$X_{6}$-Not participate	−0.194 (*)	0.079			

Note: (.) p<0.10; (*) p<0.05; (**) p<0.01; (***) p<0.001.

**Table 6:** Linear regression analysis for socio-cultural impacts and socio-demographic variables.

	Predictors	β	$SE_{\beta }$	$R^{2}$	$\mathrm{Adjusted}\;R^{2}$	F Test Value
$Y_{2}^{+}$	$X_{1}$. -Female	−1.215 (*)	0.766	0.0447	0.0224	1.998 (*)
	$X_{5}$-Official Agent	2.721 (.)	1.615			
	$X_{6}$. -Not participate	−2.064 (**)	0.796			
$Y_{2}^{-}$	$X_{1}$-Female	-0.727	0.707	0.0962	0.075	4.539 (***)
	$X_{3}$-Beyond high school	5.733 (***)	1.24			
	$X_{5}$-Fishermen	−1.791 (.)	1.035			
	$X_{5}$-Official Agent	−6.817 (***)	1.49			
	$X_{5}$-Trade and Tourism	−3.105 (**)	0.966			
	$X_{6}$-Not participating in a social organization	0.623	0.735			
$Y_{2}\;$	$X_{2}$-Single	−0.36 (*)	0.161	0.0353	0.0229	2.839 (*)
	$X_{3}$	0.001	0.003			
	$X_{6}$-Not participating in a social organization	−0.175 (*)	0.076			

Note: (.) p<0.10; (*) p<0.05; (**) p<0.01; (***) p<0.001.

**Table 7:** Linear regression analysis for environmental impacts and socio-demographic variables.

	Predictors	β	$SE_{\beta }$	$R^{2}$	$\mathrm{Adjusted}\;R^{2}$	F Test Value
$Y_{3}^{+}$	$X_{2}$-Single	−1.07 (***)	0.371	0.0971	0.0782	5.147 (***)
	$X_{3}$-Beyond high school	−0.721 (*)	0.3			
	$X_{5}$-Fishermen	0.481 (*)	0.031			
	$X_{5}$-Others	0.757 (**)	0.281			
	$X_{5}$-Trade and Tourism	0.428 (.)	0.224			
$Y_{3}^{-}$	$X_{1}$-Female	−3.1 (***)	0.752	0.1693	0.1498	8.694 (***)
	$X_{3}$-Secondary	−2.824 (**)	0.894			
	$X_{3}$-High school	−2.26 (***)	1.148			
	$X_{5}$-Official Agent	−2.709 (.)	1.585			
	$X_{5}$-Others	−3.305 (**)	1.228			
	$X_{6}$-Non socially participating	−3.156 (***)	0.781			
$Y_{3}$	$X_{1}$-Female	0.377 (***)	0.101	0.1988	0.1779	9.506 (***)
	$X_{2}$-Single	−0.652 (**)	0.224			
	$X_{3}$-High school	0.623 (**)	0.155			
	$X_{5}$-Others	0.381 (*)	0.17			
	$X_{6}$-Non socially participating	0.413 (***)	0.106			

Note: (.) p<0.10; (*) p<0.05; (**) p<0.01; (***) p<0.001.

**Table 8:** Relevant factors influencing Ly Son residents’ perceptions on tourism’s impacts.

	More Favorable	Less Favorable
Economic impacts	Married Secondary Trade and Tourism Social network	Single High-school level or Beyond Free labor No Social network
Socio-cultural impacts	Male, Married Social network Trade – Tourism service, Officials, Fishermen	Female, Single No social network Beyond High-school level
Environmental impacts	Married Female No social network Secondary, High school Free labors, Fishermen	Single Male Social network Beyond high-school level

## References

[1] Li W., Lin L., Shi-rong T., Song L., Zhao Y., Yong W., Dong-dong L. (2004). Residents’ attitudes to tourism development in ancient village resorts Case study of World Cultural Heritage of Xidi and Hong villages. Chin. Geogr. Sci..

[2] Sroypetch S. (2016). The mutual gaze: Host and guest perceptions of socio-cultural impacts of backpacker tourism: A case study of the Yasawa Islands, Fiji. Mar. Isl. Cult..

[3] Ng S.I., Chia K.W., Ho J.A., Ramachandran S. (2017). Seeking tourism sustainability—A case study of Tioman Island, Malaysia. Tour. Manag..

[4] Lv X., Li C., McCabe S. (2020). Expanding theory of tourists’ destination loyalty: The role of sensory impressions. Tour. Manag..

[5] Robinson D., Newman S.P., Stead S.M. (2019). Community perceptions link environmental decline to reduced support for tourism development in small island states: A case study in the Turks and Caicos Islands. Mar. Policy.

[6] Rasoolimanesh S.M., Ali F., Jaafar M. (2018). Modeling residents’ perceptions of tourism development: Linear versus non-linear models. Destin. Mark. Manag..

[7] Almeida-García F., Peláez-Fernández M.A., Balbuena-Vázquez A., Cortés-Macias R. (2016). Residents’ perceptions of tourism development in Benalmádena (Spain). Tour. Manag..

[8] Sdrali D., Goussia-Rizou M., Kiourtidou P. (2015). Residents’ perception of tourism development as a vital step for participatory tourism plan: A research in a Greek protected area. Environ. Dev. Sustain..

[9] Sita S.E.D., Nor N.A.M. (2015). Degree of contact and local perceptions of tourism impacts: A case study of homestay programme in Sarawak. Procedia Soc. Behav. Sci..

[10] Naidoo P., Sharpley R. (2016). Local perceptions of the relative contributions of enclave tourism and agritourism to community well-being: The case of mauritius. Destin. Mark. Manag..

[11] Garau-Vadell J.B., Gutierrez-Taño D., Diaz-Armas R. (2018). Economic crisis and residents’ perception of the impacts of tourism in mass tourism destinations. Destin. Mark. Manag..

[12] Sun Y., Cruz M.J., Min Q., Liu M., Zhang L. (2013). Conserving agricultural heritage systems through tourism: Exploration of two mountainous communities in China. Mt. Sci..

[13] Zhang Y., Zhang J., Zhang H., Zhang R., Wang Y., Guo Y., Wei Z. (2017). Residents’ environmental conservation behaviour in the mountain tourism destinations in China: Case studies of Jiuzhaigou and Mount Qingcheng. Mt. Sci..

[14] Diaz-Farina E., Díaz-Hernández J.J., Padrón-Fumero N. (2020). The contribution of tourism to municipal solid waste generation: A mixed demand-supply approach on the island of Tenerife. Waste Manag..

[15] Chiu H.Y., Chan C.S., Marafa L.M. (2016). Local perception and preferences in nature tourism in Hong Kong. Tour. Manag. Perspect..

[16] Lee T.H., Jan F.H. (2019). Can community-based tourism contribute to sustainable development? Evidence from residents’ perceptions of the sustainability. Tour. Manag..

[17] Liang Z.X., Hui T.K. (2016). Residents’ quality of life and attitudes toward tourism development in China. Tour. Manag..

[18] Bimonte S., Faralla V. (2016). Does residents’ perceived life satisfaction vary with tourist season? A two-step survey in a Mediterranean destination. Tour. Manag..

[19] Wassler P., Nguyen T.H.H., Mai L.Q., Schuckert M. (2019). Social representations and resident attitudes: A multiple-mixed-method approach. Ann. Tour. Res..

[20] Zhang H., Xu H. (2019). Impact of destination psychological ownership on residents’ “place citizenship behavior”. Destin. Mark. Manag..

[21] Huang K., Pearce P. (2019). Visitors’ perceptions of religious tourism destinations. Destin. Mark. Manag..

[22] Stylidis D., Biran A., Sit J., Szivas E.M. (2014). Residents’ support for tourism development: The role of residents’ place image and perceived tourism impacts. Tour. Manag..

[23] Šegota T., Mihalič T., Kuščer K. (2017). The impact of residents’ informedness and involvement on their perceptions of tourism impacts: The case of bled. Destin. Mark. Manag..

[24] Woosnam K.M., Draper J., Jiang J.K., Aleshinloye K.D., Erul E. (2018). Applying self-perception theory to explain residents’ attitudes about tourism development through travel histories. Tour. Manag..

[25] Boley B.B., McGehee N.G., Perdue R.R., Long P. (2014). Empowerment and resident attitudes toward tourism: Strengthening the theoretical foundation through a Weberian lens. Ann. Tour. Res..

[26] Vargas-Sánchez A., Oom do Valle P., da Costa Mendes J., Silva J.A. (2015). Residents’ attitude and level of destination development: An international comparison. Tour. Manag..

[27] Kang S.K., Lee J. (2018). Support of marijuana tourism in Colorado: A residents’ perspective using social exchange theory. Destin. Mark. Manag..

[28] Choi Y.H., Song H., Wang J.H., Hwang J. (2019). Residents’ perceptions of the impacts of a casino-based integrated resort development and their consequences: The case of the Incheon area in South Korea. Destin. Mark. Manag..

[29] Nunkoo R., Gursoy D. (2012). Residents’ support for tourism. Ann. Tour. Res..

[30] Ouyang Z., Gursoy D., Sharma B. (2017). Role of trust, emotions and event attachment on residents’ attitudes toward tourism. Tour. Manag..

[31] Wang J., Liu-Lastres B., Ritchie B.W., Pan D.Z. (2019). Risk reduction and adventure tourism safety: An extension of the risk perception attitude framework (RPAF). Tour. Manag..

[32] Vincenzi S.L., Possan E., de Andrade D.F., Pituco M.M., de Santos T.O., Jasse E.P. (2018). Assessment of environmental sustainability perception through item response theory: A case study in Brazil. Clean. Prod..

[33] Yan L., Gao B.W., Zhang M. (2017). A mathematical model for tourism potential assessment. Tour. Manag..

[34] Sheppard V.A., Williams P.W. (2016). Factors that strengthen tourism resort resilience. Hosp. Tour. Manag..

[35] Kim K., Uysal M., Sirgy M.J. (2013). How does tourism in a community impact the quality of life of community residents?. Tour. Manag..

[36] Moghavvemi S., Woosnam K.M., Paramanathan T., Musa G., Hamzah A. (2017). The effect of residents’ personality, emotional solidarity, and community commitment on support for tourism development. Tour. Manag..

[37] Jordan E.J., Spencer D.M., Prayag G. (2019). Tourism impacts, emotions and stress. Ann. Tour. Res..

[38] Blieszner R., Roberto K.A., Singh K. (2001). The helping networks of rural elders: Demographic and social psychological influences on service use. Ageing Int..

[39] Fayos G.T., Gallarza G.M.G., Arteaga M.F., Elena F.I. (2014). Measuring socio-demographic differences in volunteers with a value-based index: Illustration in a mega event. Voluntas.

[40] Mak W., Rosenblatt A. (2002). Demographic influences on psychiatric diagnoses among youth served in California systems of care. Child Fam. Stud..

[41] Van der Steina A., Rozite M., Müller D., Więckowski M. (2018). Tourism development in Riga: Resident attitudes toward tourism. Tourism in Transitions. Geographies of Tourism and Global Change.

[42] Yergeau M.E. (2020). Tourism and local welfare: A multilevel analysis in Nepal’s protected areas. World Dev..

[43] Develles R.F. (1991). Scale Development: Theory and Applications.

[44] Kline P. (2000). The Hand$book of Psychological Testing.

[45] Pardoe I. Multiple Linear Regression Model Evaluation (Lecture 6); In STAT 501 Online Course; Department of Statistics, Eberly College of Science. https://online.stat.psu.edu/stat501/lesson/6/6.2.

[46] Ramachandran K.M., Tsokos C.P. (2009). Mathematical Statistics with Applications.

[47] Zar J.H., Lynch D., O’Brien C. (2010). Two-factor analysis of variance. Biostatistical Analysis.

[48] LSGOS (General Statistics Office of Ly Son) (2018). Statistical Yearbook of Ly Son District 2018.

